# Inter-rater Reliability of the Ramsay Sedation Scale for Critically-ill Intubated Patients

**DOI:** 10.7759/cureus.6021

**Published:** 2019-10-29

**Authors:** Harmeet S Deol, Salim R Surani, George Udeani

**Affiliations:** 1 Miscellaneous, Corpus Christi Rehabilitation Hospital, Corpus Christi, USA; 2 Internal Medicine, Texas A&M Health Science Center, Temple, USA; 3 Miscellaneous, Corpus Christi Cancer Center, Corpus Christi, USA

**Keywords:** ramsay, sedation, scale, interdisciplinary

## Abstract

Introduction: Titratable palliative sedation (TPS) is frequently applied in sedative therapeutics to ameliorate unendurable and refractory distress via reduction in patient consciousness. TPS may be adjusted based on objective and subjective data: vitals, labs, Ramsay Sedation Scale (RSS), and Richmond Agitation-Sedation Scale (RASS). Inappropriate dosing, including over-sedation from variability in clinician assessment of sedation scales, can contribute to significant negative clinical outcomes. We evaluated inter-rater reliability (IRR) and its relationship to variations in dosing to determine whether additional training in sedation scale assessment is necessary at our community institution.

Methods: This was a prospective study assessing sedation in intensive care unit (ICU) mechanically ventilated patients without neurogenic abnormalities. Non-nursing healthcare personnel conducted independent sedation assessments using the RSS and compared their evaluations to those documented by the nursing staff. Data obtained from the patients' chart included: demographics, Ramsay Score, past medical history, diagnosis, and body mass index (BMI). Post-analysis, non-nursing healthcare personnel scores were classified into three categories: equal to, higher than, or lower than those charted by nursing staff.

Results: There were 83 random RSS assessments conducted in 44 patients with a mean age of 63.6 +/10.09 years (range: 38-82) and a mean BMI of 31.2 +/12.4 (range: 15-77). 19/42 (45%) patients had a diagnosis of respiratory failure or pneumonia. Other diagnoses included congestive heart failure (3), seizures (5), aortic valve replacement (1), small bowel obstruction (1), drug overdose (2), cardiac arrest (2), and urinary tract infection (1), ST-elevated myocardial infarction (2), pulmonary embolism (2), coronary artery bypass graft (1), sepsis (1), hemoptysis (1), altered mental status (1). Non-nursing healthcare professionals' assessments were compared to nurses' and observed to be equal in 29%, higher in 59%, and lower in 12% of the cases. Of the 83 assessments, the average RSS score non-nursing healthcare professionals assigned was 4.8 +/1.6 while the nurses' charted average was3.39 +/- 0.97; a mean difference of 1.45, 95% CI (1.04 - 1.85)p< 0.0001.

Conclusions: Our data demonstrated equal RSS ratings in only 29% of cases for non-nursing healthcare personnel and nurses’ evaluations. Without proper education, the RSS may not be a reliable tool for sedation assessments and may result in over-sedation of critically ill patients. Recurrent nursing education is warranted to ensure proper use and optimization of the RSS.

## Introduction

In critically ill patients, specifically those in intensive care units (ICUs), pharmacologic therapy is a vital component to achieve titratable sedation and hypnosis via palliative sedation therapy, allowing clinicians to establish baseline characteristics in otherwise unstable patients [1]. Palliative sedation therapy is defined as “the use of sedative medications to relieve intolerable and refractory distress by the reduction in patient consciousness” [2]. Continuous deep sedation may be indicated in patients for several reasons, which may include reduction of intracranial pressure, intubation, seizure control, and complications of surgical intervention or trauma [3]. Adherence to institutional sedation guidelines is often low, and patients are deeply sedated, leading to sedation-related adverse events [4].

Titration of sedative therapy is generally based on both objective and subjective data (Ramsay Sedation Scale (RSS) rating, and Richmond Agitation-Sedation Scale (RASS) have been employed). The subjective component can be translated to an objective numerical score, which is used for drug therapy and dosage modification. This score certainly can have a profound influence on sedative dosing. The focus of this study will be to assess and analyze subjective data that clinicians use to guide the dosing of sedative medications.

Commonly used agents for the induction of deep sedation include propofol, midazolam, and lorazepam. The choice of agent selected can be influenced by pharmacodynamics and pharmacokinetics, as well as patient-specific medical conditions.

Assessing inter-rater reliability (IRR) can be a necessity in research studies that are designed to collect data from “trained or untrained coders”. IRR assessment allows quantification of the level of agreement amongst independent raters. We attempted to determine the level of agreement amongst healthcare professionals that assess patients under critical care, using IRR. IRR relies on the computation of variability to determine reliability amongst various raters. In this study, the coders were the healthcare staff that were evaluating a patient using the RSS.

This study was an observational study using the RSS ratings, conducted by multiple coders (nurses and independent physicians and pharmacists). Since the objective of this study is to evaluate whether nursing and other healthcare staff are appropriately rating sedated patients; using the RSS we cannot control the training that the staff receive. In order to verify the measurements taken by the nursing staff we, however, used the independent coders who were trained to establish baseline competency in using RSS.

This study was a pilot study to evaluate feasibility, time, and effort in order to scale the study in case an effect is observed. We expect the nursing staff and other such raters to have valid RSS assessments of 65% of the time and physicians and pharmacists to be valid in 90% of their assessments. With an alpha value of 0.05 and with an 80% power we need a sample size of at least 43 patients to detect a difference in validity and assess reliability [5].

## Materials and methods

This entailed a prospective data collection study at our institution, which is a community hospital with an internal medicine residency program [1]. This study was conducted in the area of critical care, specifically focusing on deep sedation; patients were in the ICU environment and the clinical data collected included drug name and dose, if the patient was being administered midazolam, lorazepam, or propofol. Vitals and labs were collected with no impact on patient treatment choices. Demographic data included race, age, sex, weight, height, comorbidities, and past medical history [1]. ICU nurses were blinded to the study as this was a quality control study and the nurses’ scores were collected directly from the chart. The RSS scores the nurse determined and documented were collected, as well as interventions to increase and decrease the sedative dose. Another healthcare profession (physician or pharmacist) also carried out the above activities on a given patient independently of nursing interventions and without referencing nursing scores prior to assessments. The pharmacist and physician assessments were performed randomly and their scores were then compared with the scores documented in the patient chart by nursing staff [1].

Patients were assigned random numbers, to protect their identity upon enrollment, throughout the entire study. Data were analyzed by the investigators, to determine variability in Ramsay scores by various healthcare professionals.

The study subjects included the patients admitted to our institution who were receiving sedatives. Multivariable data analysis was done to determine IRR and differences in interdisciplinary scoring of the RSS [1].

## Results

**Figure 1:**
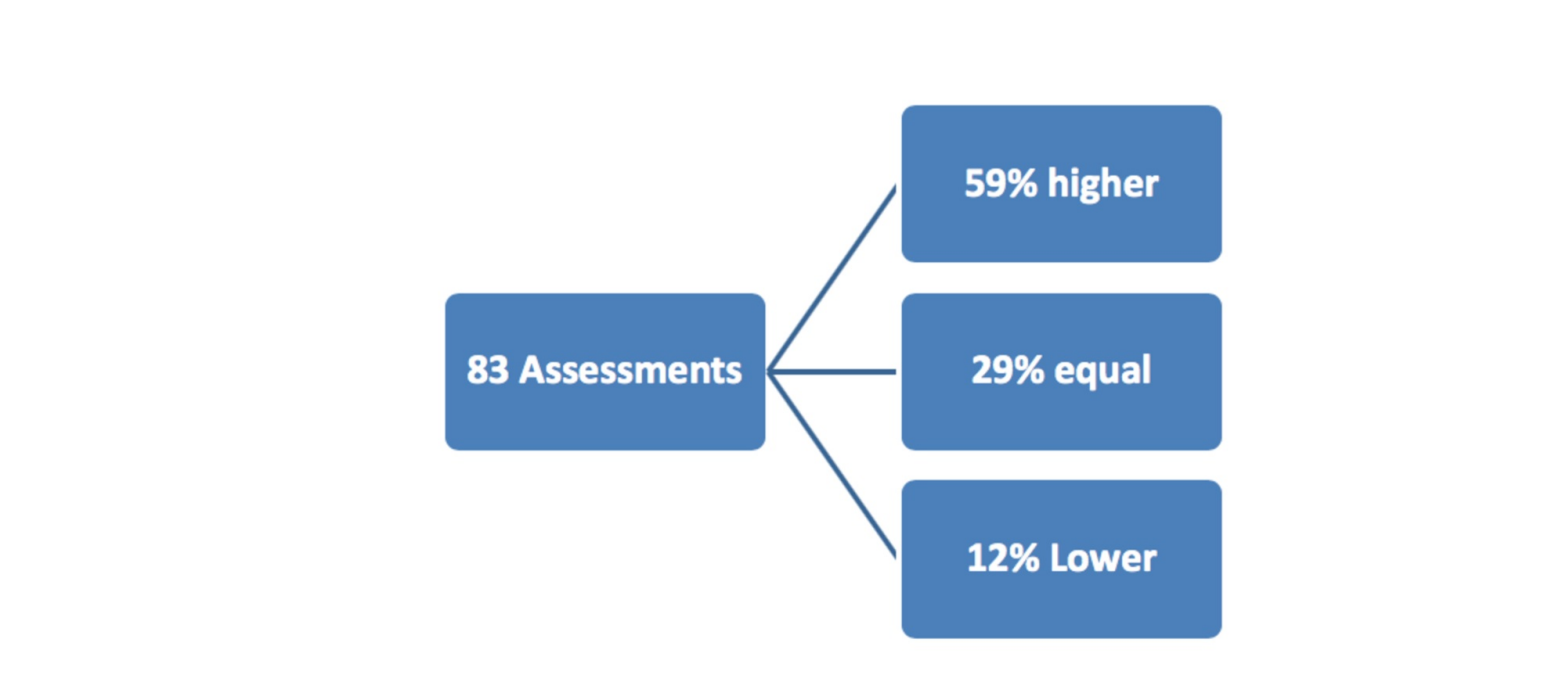
Comparison of nurses Ramsay Sedation Scale assessments to other health care professionals assessments

**Figure 2:**
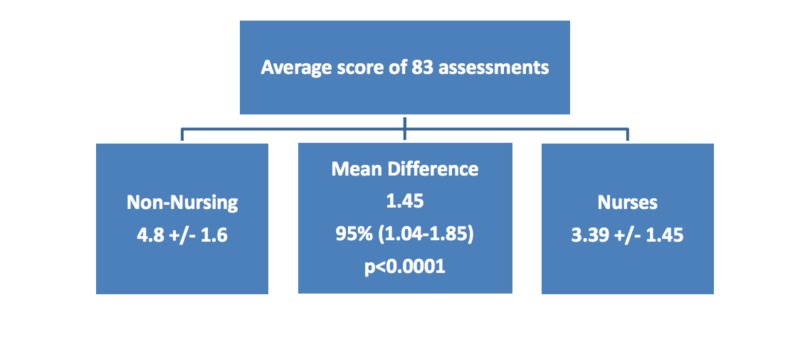
Comparison of interdisciplinary scoring of the Ramsay Sedation Scale

There were 83 random RSS assessments conducted in 44 patients with a mean age of 63.6 +/- 10 years (range: 38-82) and a mean body mass index (BMI) of 31.2 +/12.4 (range: 15-77) [1]. Nineteen (45%) of the 42 patients had a diagnosis of respiratory failure or pneumonia. Other diagnoses included congestive heart failure (3), seizures (5), aortic valve replacement (1), small bowel obstruction (1), drug overdose (2), cardiac arrest (2), and urinary tract infection (1), ST-elevation myocardial infarction (2), pulmonary embolism (2), atherosclerosis requiring coronary artery bypass graft (1), sepsis (1), hemoptysis (1), altered mental status (1) (Table 1). Non-nursing healthcare professionals' assessments were compared to nurses' and observed to be equal in 29%, higher in 59%, and lower in 12% of the cases [1]. Of the 83 assessments, the average RSS score non-nursing healthcare professionals assigned was 4.8 +/- 1.6 while the nurses' charted average was 3.39 +/- 0.97; a mean difference of 1.45, 95% CI (1.04 - 1.85) p< 0.0001 [1] (Figures 1-2).

**Table 1:** Primary diagnosis of intubated and sedated patients

Diagnosis	Number of Patients
Respiratory failure/ pneumonia	19
Seizure	5
Congestive heart failure	3
Drug overdose	2
Drug Overdose	2
Cardiac arrest	2
ST-Elevation Myocardial Infarction	2
Pulmonary embolism	2
Atherosclerosis requiring coronary artery bypass graft	1
Sepsis	1
Hemoptysis	1
Altered mental status	1
Urinary tract infection	1
Aortic valve replacement	1
Small bowel obstruction	1
Total	44

## Discussion

Studies have demonstrated the reliability of the RSS. Our analysis indicated there is disagreement amongst healthcare professionals of various disciplines when using the RSS to assess sedation. At one point during the study, the results of both the non-nursing and nursing scores were in constant agreement. We believe this was a direct result of the nurses observing the investigating team regularly collecting data from the charts. This occurrence led the investigating team to stop collecting data for a week in order to prevent any observational bias from affecting the nursing practices. When resumed, the data collection again produced results that were consistent with the normal data recording patterns first observed. This incidence leads us to believe that, other than lack of training, there may be other factors associated with the use of the Ramsay score that may be influencing inappropriate use of the scale.

A 2008 publication Mechanical Ventilation highlights the shortcomings of the RSS when applied in the ICU patients who may have a sluggish response to stimuli, yet still be restless and anxious; this subjective variability is common as patients may fall into more than one level of sedation when using the scale [6]. According to the authors, the simplicity of the scale makes it a desirable assessment tool in the ICU. This may be a confounding factor that was not accounted for in our study. The pharmacist and physician assessments were performed in random and their scores were then compared with the scores documented in the patient chart by nursing staff, so it is very possible for nursing and non-nursing assessors to rate the patient at different levels of sedation. A 2018 publication Essentials of Pain Medicine also highlights the simplicity of the RSS which makes it a good choice to use in the ICU [7] The authors note that unlike the Riker Sedation-Agitation Scale (SAS), which allows the patients sedation level to be scored to highlight different extremes of sedation, the RSS’s subjective nature makes it difficult for the rater to differentiate between various levels of sedation.

Time constraints and other nursing-related duties and lack of appropriately trained staff may also be contributors to the improper utilization of the RSS. We did not assess the training provided to nurses working in the ICU prior to the beginning of the study. The nurses had various levels of experience working in the ICU and prior use of the scale was not confirmed; competency was assumed since basic training on the use of the RSS is provided to all nurses in the ICU. Hands-on training in using the scale is recommended in a multidisciplinary setting. 

The validity and reliability of the RSS have been confirmed in other studies; one such study performed in ICUs in Iran and Brazil concluded the RSS is a reliable and valid tool for assessing sedation [8-9]. An article published in 2006 in a Scandinavian Anesthesia Journal also found variability in RSS assessments and found that adherence to the hospital guidelines for titration and tapering of sedatives based on RSS findings was low [10]. This study also implicated the continuous need for training, education, and discussion.

## Conclusions

Findings of our study highlight that without proper training and education, the RSS may have significant inter-rater variability associated with its use. We suggest institutions periodically set and assess competencies and training for staff using the RSS. A multidisciplinary approach to sedation assessment provides an opportunity to identify areas of improvement such as education and training on the practical implementation of the RSS. The RSS allows for significant subjective variability amongst assessors, as many other studies have confirmed the reliability of the RSS. The caveat our study highlights is that inter-rater variability will occur if appropriate training and education is not provided to healthcare professionals utilizing the RSS.
